# MFS transportome of the human pathogenic yeast *Candida albicans*

**DOI:** 10.1186/1471-2164-9-579

**Published:** 2008-12-03

**Authors:** Manisha Gaur, Nidhi Puri, Raman Manoharlal, Versha Rai, Gauranga Mukhopadhayay, Devapriya Choudhury, Rajendra Prasad

**Affiliations:** 1 Special Centre for Molecular Medicine, Jawaharlal Nehru University, New Delhi, India; 2 School of Life Sciences, Jawaharlal Nehru University, New Delhi, India; 3 School of Biotechnology, Jawaharlal Nehru University, New Delhi, India; 4 School of Information Technology, Jawaharlal Nehru University, New Delhi, India

## Abstract

**Background:**

The major facilitator superfamily (MFS) is one of the two largest superfamilies of membrane transporters present ubiquitously in bacteria, archaea, and eukarya and includes members that function as uniporters, symporters or antiporters. We report here the complete transportome of MFS proteins of a human pathogenic yeast *Candida albicans*.

**Results:**

Computational analysis of *C. albicans *genome enabled us to identify 95 potential MFS proteins which clustered into 17 families using Saier's Transport Commission (TC) system. Among these SP, DHA1, DHA2 and ACS represented major families consisting of 22, 22, 9 and 16 members, respectively. Family designations in *C. albicans *were validated by subjecting *Saccharomyces cerevisiae *genome to TC system. Based on the published available genomics/proteomics data, 87 of the putative MFS genes of *C. albicans *were found to express either at mRNA or protein levels. We checked the expression of the remaining 8 genes by using RT-PCR and observed that they are not expressed under basal growth conditions implying that either these 8 genes are expressed under specific growth conditions or they may be candidates for pseudogenes.

**Conclusion:**

The *in silico *characterisation of MFS transporters in *Candida albicans *genome revealed a large complement of MFS transporters with most of them showing expression. Considering the clinical relevance of *C. albicans *and role of MFS members in antifungal resistance and nutrient transport, this analysis would pave way for identifying their physiological relevance.

## Background

Current evidence suggests that *Candida albicans *acquires azole resistance by employing multiple mechanisms that include (a) alterations in the azole-target protein Erg11p (b) upregulation of the *ERG11 *gene [1-4] as well as (c) failure of drug accumulation mediated by efflux pumps. Most commonly, genes encoding drug efflux pumps belonging to ATP binding cassette (ABC) and Major facilitator (MFS) superfamilies of proteins are overexpressed in azole resistant *Candida *isolates [5-9]. ABC family permeases are in general multicomponent primary active transporters, capable of transporting both small molecules and macromolecules which is coupled to ATP hydrolysis while the MFS transporters are single-polypeptide secondary carriers capable only of transporting small solutes in response to chemiosmotic ion gradients. We have earlier annotated and classified ABC transporters in *C. albicans *[10], however, clinically relevant MFS superfamily in *C. albicans *largely remains uncharacterized.

MFS superfamily is ubiquitously present in all kingdoms of life and includes members of direct medical and pharmaceutical significance. They are involved in the symport, antiport or uniport of various substrates [11,12] and are known to exhibit specificity for sugars, polyols, drugs, neurotransmitters, Krebs cycle metabolites, phosphorylated glycolytic intermediates, amino acids, peptides, osmolites, siderophores (efflux), iron-siderophores (uptake), nucleosides, organic and inorganic anions, etc [11,12]. Most MFS proteins vary between 400 and 600 amino acid residues in length and possess either 12 or 14 putative transmembrane segments (TMS). The MFS superfamily consists of 61 families according to the Transport Commission (TC) system given by Saier and group . TC is a comprehensive classification system for membrane transport proteins and is analogous to the Enzyme Commission (EC) system, except that it incorporates both functional and phylogenetic information [13-15]. This system allocates five digits to each phylogenetic cluster of transporters. The first two digits ("class" and "subclass") identify the transport mode and energy-coupling mechanism. The third digit characterizes phylogenetic "families" or "superfamilies." The fourth digit identifies phylogenetic "subfamilies." The fifth digit ("clusters") corresponds to the substrate specificity, as presumed by experimental data or stringent sequence identity [13]. In TC, the designation 2.A.1 represents MFS and the next digit denotes the family, for instance, 2.A.1.1 represents sugar transporters and so on. Any two transport systems in the same subfamily of a transporter family that transport the same substrate(s) are given the same TC number, regardless of whether they are orthologues or paralogues.

Till date only a few MFS transporters namely *MDR1*, *FLU1*, *NAG3*, *NAG4*, *JEN1*, *ARN1 *and *NGT1 *[16-22], have been identified and characterized in *C. albicans*. Additionally, over 20 hexose transporters and glucose sensors are known to exist in *C. albicans *that reflect the varied niches in which this pathogen thrives [23]. However, very limited knowledge about other MFS transporters is available in *C. albicans*. Out of all the known MFS in *C. albicans MDR1*, its alleles and *FLU1 *are shown to be the only drug efflux pumps transporters. *MDR1 *was initially identified as a gene, which conferred resistance to the tubulin binding agent benomyl and tetrahydrofolate reductase inhibitor methotrexate [24,25]. *MDR1 *expression in *S. cerevisiae *confers resistance to several unrelated drugs and its overexpression has been linked to azole resistance in *C. albicans*. The expression of *MDR1 *in *C. albicans *cells is enhanced by benomyl, methotrexate and several other unrelated drugs, and is found to be more pronounced in some of the azole resistant clinical isolates [26,27]. Keeping in view, the relevance of the MFS multidrug transporters in general and in multidrug resistance (MDR) in particular, in the present study, we have examined MFS superfamily of proteins in *C. albicans*. Although, earlier annotation of the previous *Candida *genome assembly (version 19) predicted 71 MFS genes, no systematic classification was given [28]. To address this question, we have applied a comprehensive bioinformatics approach to identify and annotate all MFS transporter genes in the *Candida *genome from assembly version 21 and systematically searched for evidence of their expression. It is hoped that these findings will provide the scientific community with the necessary framework needed for the functional characterization of the MFS proteins and a better understanding of this medically and pharmaceutically significant superfamily.

## Results

### Identification and sequence-based functional grouping of *C. albicans *putative MFS genes

**Figure 1:**
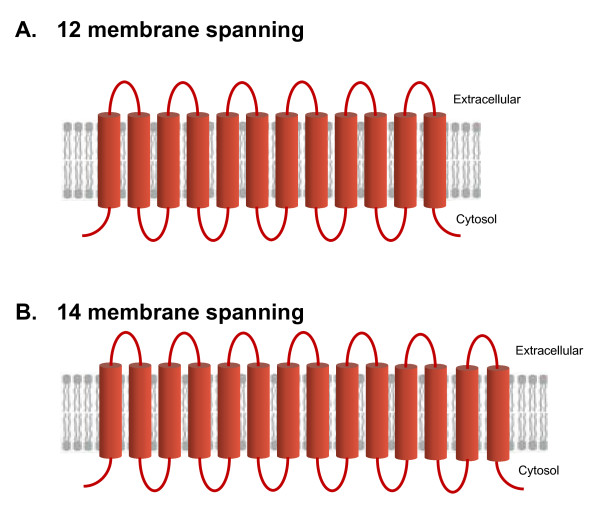
**Predicted topology of putative MFS proteins of *C. albicans***. The topology of the putative MFS proteins was predicted using TMHMM program . The transmembrane domains were found to be either 12 or 14 transmembrane spanners.

To identify gene loci encoding MFS proteins, multiple TBLASTN searches were performed on assembly (version 21) of the *C. albicans *genome  using well known MFS proteins as queries. The ORFs thus identified were subjected to domain analysis followed by sequence-based functional grouping, resulting in clustering of putative *Candida *proteins into various MFS families as described by Saier's TC system. Using this strategy, we identified a total of 95 loci of putative MFS genes in the *Candida *genome containing the domains characteristic of MFS proteins and were found to be either 12 or 14 transmembrane spanners (Figure 1). By using TC system, all the putative MFS identified were assigned to 17 TC families (Additional file 1). The family assignment obtained by this approach was validated by applying the same strategy to *S. cerevisiae *MFS transportome (data not shown). *S. cerevisiae *homologues of *C. albicans *MFS proteins were found to take up the same TC cluster thereby addressing the evolutionary relationship between the two yeasts. A summary of the previously known *Candida *genes together with the new genes is presented in Additional file 1, where, TC family, CGD ORF, gene, alias, TCDB homolog and expression confirmation are listed. In addition, the closest *S. cerevisiae *member within the family is also presented in Additional file 1.

### The Sugar Porter (SP) Family (TC # 2.A.1.1)

The SP family is widespread and have members from all of the major groups of living organisms: bacteria, archaea, eukaryotic protists, fungi, animals and plants [11]. It forms one of the largest families with 22 members in *C. albicans*. We have previously characterized *HGT1 *[29] and many studies since then have also reported other SP members namely *HGT2-HGT20 *[23,30]. Apart from identifying *HGT1-HGT20*, in the present study, we have also identified two previously unidentified members, namely *MAL31 *(orf19.3981) and orf19.4923 (Additional file 1) both having homologues in *S. cerevisiae*, *MAL31 *and YFL040W, respectively. Further, based on the homology, the 22 SP members show significant similarity with various sugars, namely arabinose, quinate, myoinositol, maltose, fructose, glycerol, monosaccharide, glucose, hexoseand xylose (Additional file 1).

### The Drug: H^+ ^Antiporter-1 (DHA1) Family (TC # 2.A.1.2)

DHA1 family like the SP is widely distributed and the members include both drug-specific and MDR efflux pumps. Like SP it also forms one of the largest families having 22 members in *Candida *(Additional file 1). *MDR1 *gene in *C. albicans *is one of the best characterized members, originally known to confer resistance to benomyl and methotrexate [19,20,24,25]. Subsequent studies indicated that *MDR1 *also encodes resistance to cycloheximide, benztriazoles, 4-nitroquinolone-*N*-oxide and sulfometuron methyl [24,31]. Disruption of the *MDR1 *gene reduced the virulence of *C. albicans *[26]. Other characterized members of this family include *FLU1*, *NAG3 *and *NAG4*. Disruption of *FLU1 *in *C. albicans *hyper-susceptibility to mycophenolic acid thus suggesting that it could be a preferred substrate for the transporter [17]. On the other hand, *NAG3 *(*TMP1*) and *NAG4 *(*TMP2*) show susceptibility to a number of unrelated compounds such as cycloheximide, 4-nitroquinoline-*N*-oxide and 1,10-phenanthroline and are upregulated in response to these drugs, suggesting that they function as multiple drug efflux pumps [21]. Apart from *MDR1*, which is known as a clinically relevant efflux pump protein, none of the other characterized members have been directly linked to MDR of *C. albicans*.

### The Drug: H^+ ^Antiporter-2 (DHA2) Family (TC # 2.A.1.3)

The DHA2 family of drug:H^+ ^antiporters with 14 predicted transmembrane-spanning segments, consists of nine members in *C. albicans *which show significant similarity to transporters, namely aminotriazole, 4-nitroquinoline-*N*-oxide, Me^2+^-tetracycline antiporter, vacuolar basic amino acid (Arg, Lys, His) transporter and metal:tetracycline/oxytetracycline efflux pump (Additional file 1). In *C. albicans *no member of this family has yet been characterized whereas in *S. cerevisiae *two DHA2 proteins, *SGE1 *and *ATR1 *are well studied. *ATR1 *has been shown to confer resistance to the structurally unrelated compounds aminotriazole and 4-nitroquinolone-*N*-oxide and expression of *ATR1 *is inducible by the former but not the latter [32,33]. *SGE1 *appears to confer resistance to crystal violet [34] and ethidium bromide [35,36].

### The Fucose: H^+ ^Symporter (FHS) Family (TC # 2.A.1.7)

FHS is a small family with two ORFs identified in *Candida *namely: orf19.4090 and orf19.7490 (Additional file 1). They are homologous to *S. cerevisiae BSC6*, which encodes a protein of unknown function exhibiting genomic organization compatible with a translational read through-dependent mode of expression [37].

### The Phosphate: H^+ ^Symporter (PHS) Family (TC # 2.A.1.9)

PHS family is unusual in that it has representatives only in yeast, fungi and plants but none in bacteria, animals and other eukaryotes [11]. The occurrence of distant homologues in both the plant and fungal kingdoms suggests that they possess isoforms that diverged from each other well before plants diverged from fungi [11]. Two well characterized members of the PHS family are the Pho84 inorganic phosphate transporter of *S. cerevisiae *[38] and the GvPT phosphate transporter of *Glomus versiforme *[39]. In this study, we have identified five ORFs belonging to PHS family in *C. albicans *with homology to phosphate: H^+ ^symporters (Additional file 1). However, none of the members identified in *Candida *has yet been characterized.

### The Oxalate: Formate Antiporter (OFA) Family (TC # 2.A.1.11)

OFA family members are widely distributed in nature, being present in the bacterial, archaeal and eukaryotic kingdoms [11]. In *C. albicans*, our searches revealed two members (Additional file 1). OxlT, the oxalate:formate antiporter from *Oxalobacter formigenes*, is the hallmark protein and provides the basis for naming the OFA family [40,41]. This protein has been purified, reconstituted in an artificial membrane system as well as structurally and functionally characterized [42,43].

### The Sialate: H^+ ^Symporter (SHS) Family (TC # 2.A.1.12)

SHS family, like the PHS family, is very small with only two members namely *JEN1 *and *JEN2*, identified in the present as well as a previous study [44]. *JEN1 *has been described as the first monocarboxylate transporter of *C. albicans *showing loss of all measurable lactate permease activity upon its disruption. Further, lactate uptake by *JEN1 *was competitively inhibited by pyruvic and propionic acids while acetic acid behaved as a non-competitive substrate [22].

### The Monocarboxylate Porter (MCP) Family (TC # 2.A.1.13)

MCP family is exclusively present in yeasts and animals. In mammals, these permeases are known to transport monocarboxylates, namely pyruvate, lactate and mevalonate with inwardly-directed polarity and presumably function as proton symporter [11] while it is reported that the yeast monocarboxylate transporter proteins perform functions other than their mammalian counterparts [45]. This family has six members identified in *Candida*.

### The Anion: Cation Symporter (ACS) Family (TC # 2.A.1.14)

ACS is a relatively large family having representation in bacteria, yeasts and animals comprising mainly of symporters that are known to accumulate their substrates in symport with either Na^+ ^or H^+^, depending on the system. They may transport either inorganic (e.g. phosphate) or organic anions (e.g. glucarate, hexuronate, tartrate, allantoate or 4-hydroxylphenyl acetate) [11]. In *Candida*, we have identified 16 members showing significant similarity to transporters having prefered substrates, namely tartrate, allantoate, nicotinate, biotin and pantothenate (Additional file 1).

### The Aromatic Acid: H^+ ^Symporter (AAHS) Family (TC # 2.A.1.15)

The members of AAHS family occur exclusively in gram-negative bacteria where they are known to transport a variety of aromatic acids like benzoate, 4-hydroxybenzoate, 3-hydroxyphenylpropionate, 2,4-dichlorophenoxyacetate as well as niacin and *cis*, *cis*-muconate [11]. In *Candida*, a single member of AAHS family has been identified, namely orf19.6952 showing significant similarity to putative niacin uptake porter (Additional file 1). This family has no representation in *S. cerevisiae *and thus unique to *Candida*.

### The Siderophore-Iron Transporter Family (TC # 2.A.1.16)

All the known members of this family are from yeast species. In *C. albicans *this family is represented by a single protein known as siderophore transporter, *SIT1/ARN1 *(orf19.2179) which is required in ferrichrome-iron uptake. Previous reports suggest that deletion of *ARN1 *leads to reduced ability of *C. albicans *to use iron bound to the hydroxamate-type siderophore ferrichrome and upon deletion of the two high-affinity iron permease *C. albicans *genes (*FTR1 *and *FTR2*), the activity was completely abolished [18,46]. According to another study, siderophore uptake by Sit1p/Arn1p is required in a specific process of *C. albicans *infection, namely epithelial invasion and penetration, while in the blood or within organs other sources of iron, including heme, may be used [47].

### The Organic Cation Transporter (OCT) Family (TC # 2.A.1.19)

In *C. albicans *this family is represented by a single uncharacterized member *FGR2 *(orf19.7071) showing similarity to organic anion: dicarboxylate transporter. These proteins are known to transport organic cations and/or anions and catalyze uptake of cationic drugs such as tetramethyl ammonium, cimetidine, procainamide, quinidine and some endogenous metabolites such as *N*-methyl-nicotinamide [48-51].

### The Vesicular Neurotransmitter Transporter (VNT) Family (TC # 2.A.1.22)

These proteins are more closely related to SP family than to other MFS families. The better characterized members of the VNT family are synaptic vesicle proteins from mammals, the electric eel and insects [52-55]. In *C. albicans *this family is represented by a single member orf19.6578 with significant similarity to dopamine transporter.

### The Peptide-Acetyl-Coenzyme A (PAT) Transporter Family (TC # 2.A.1.25)

Members of the PAT family are present across bacteria, yeast and animals [11]. Amongst the well characterized proteins of this family include acetyl-CoA transporter localized in the endoplasmic reticulum and Golgi membranes of humans [56]. AmpG protein of *E. coli *belonging to PAT family, brings into the cell peptides, including cell wall degradative peptides and glycopeptides, to act as inducers of β-lactamase synthesis [57]. The acetyl-CoA transporter is expected to function by acetyl-CoA:CoA antiport while the AmpG protein is most likely energized by substrate:H^+ ^symport. In *C. albicans *this family is represented by a single member orf19.3782 with significant homology to acetyl-CoA:CoA antiporter.

### The L-Amino Acid Transporter-3 (LAT3) Family (TC # 2.A.1.44) (also called the SLC43 family)

LAT3 transports neutral amino acids such as L-leucine, L-isoleucine, L-valine and L-phenylalanine by a Na^+^-independent, electroneutral, facilitated diffusion process and also transports amino acid alcohols. In *C. albicans*, this family is represented by two ORFs: orf19.6654 and orf19.6316.

### The Proton Coupled Folate Transporter/Heme Carrier Protein (PCFT/HCP) Family (TC # 2.A.1.50)

In *C. albicans*, this family is represented by a single member orf19.6976 showing homology to high-affinity folate transporter. PCFT from human has been shown to act both as an intestinal proton-coupled high-affinity folate transporter and as an intestinal heme transporter which mediates heme uptake from the gut lumen into duodenal epithelial cells. The iron is then released from heme and may be transported into the bloodstream [58,59].

### The N-Acetylglucosamine Transporter Family (TC # 2.A.1.58)

*NGT1 *from *C. albicans *represents the first eukaryotic *N*-acetylglucosamine (GlcNAc) transporter and is the only known member of this family. It is required for efficient GlcNAc uptake and for inducing hyphae development at low GlcNAc concentrations [16]. High concentrations of GlcNAc could bypass the need for *NGT1 *to induce hyphae, indicating that elevated intracellular levels of GlcNAc induce hyphal formation. Expression of *NGT1 *in *S. cerevisiae *promoted GlcNAc uptake, indicating that *NGT1 *acts directly as a GlcNAc transporter [16]. No homologue of *NGT1 *was detected in *S*.*cerevisiae*.

### Most of the identified members of MFS superfamily are expressed

**Figure 2:**
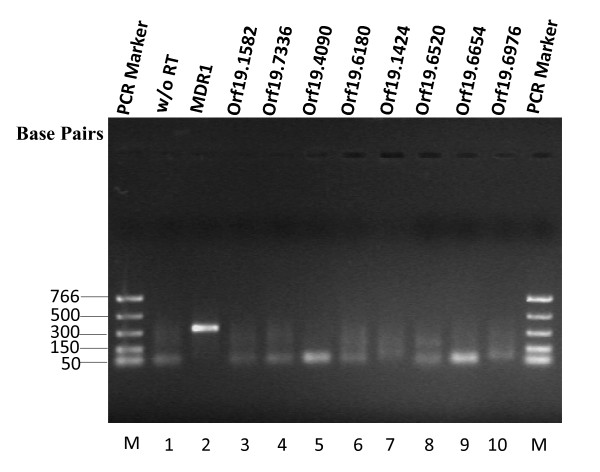
**Expression analysis of putative MFS genes by RT-PCR**. The expression of 8 putative MFS genes, which were not validated by the mRNA/protein profiling data mining, was checked by RT-PCR. Purified poly(A)^+ ^enriched mRNA fractionated from *C. albicans *isolate SC5314 were amplified by RT-PCR, as described in the *Methods*. Following electrophoresis through 1.2% agarose gel, the amplified PCR products were visualized by staining with ethidium bromide. *Lane M*, nucleotide size marker (PCR Marker); *lane 1*, without RT (negative control); *lane 2*, *MDR1 *(positive control, 330 base pairs); *lane 3*, orf19.1582; *lane 4*, orf19.7336; *lane 5*, orf19.4090; *lane 6*, orf19.6180; *lane 7*, orf19.1424; *lane 8*, orf19.6520; *lane 9*, orf19.6654 and *lane 10*, orf19.6976.

To assess which of the identified *Candida *MFS transporter genes are transcribed or translated, we analyzed all 95 MFS transporter loci by extensive mining of the data available from the genome or proteome-wide studies in *C. albicans*. Using this approach, we found that out of the 95 ORFs, 87 were shown to express either at mRNA or protein level under different experimental conditions (see Additional file 1). To validate the expression of the remaining 8 putative MFS genes (orf19.1582, orf19.7336, orf19.4090, orf19.6180, orf19.1424, orf19.6520, orf19.6654 and orf19.6976), we employed reverse transcriptase PCR (RT-PCR) approach taking a well characterized *C. albicans *MFS transporter, *MDR1 *as a positive control. The primers utilized for expression analysis are shown in Table 1. Interestingly, no expression was detected in any of the 8 putative genes tested under the basal condition (Figure 2).

**Table 1:** Oligomers used for RT-PCR

**Oligomer**	**Sequence (5'-3')**	**Expected amplicon length (bp)**
**MDR1-F**	CACCGTTATGGAACCAGTTG	330
**MDR1-R**	CAGCACCAAACAATGGACCAACCCAATGAG	
**orf19.1582-F**	GAAACTTTGGTATCCTGGAAC	380
**orf19.1582-R**	CAACAAAATGGCAAAACCACC	
**orf19.7336-F**	CGCTTTCCAACCATCAATGG	464
**orf19.7336-R**	CAGTCATTGAAGAAGCAGAAG	
**orf19.4090-F**	GAGAAGGGGCGTTTTTATTG	301
**orf19.4090-R**	CACAATGAAAACCGGTAACAC	
**orf19.6180-F**	GGTTGTTGTTAGGTGTGTTG	394
**orf19.6180-R**	CAAAATCTCGTAAACCCACG	
**orf19.1424-F**	CAGTACAAACATTACAAGCCC	476
**orf19.1424-R**	CACCACAAATGTCATACCAC	
**orf19.6520-F**	GCCTTACATCCACGCAATTTG	339
**orf19.6520-R**	CTAAAATCTAACCTCTTGGCGC	
**orf19.6654-F**	CTATTGGGTTGTTGGGTTTG	286
**orf19.6654-R**	GTCGAGCCTCCAATAATACCTG	
**orf19.6976-F**	CTCCCCCTTGGTTATATTAAC	603
**orf19.6976-R**	CCAGGCCAACCATTTTTCAAAG	

## Discussion and conclusion

In this study, we report the complete transportome of MFS superfamily of *C. albicans*. Computational analysis of the *C. albicans *genome assembly (version 21) from CGD enabled us to identify 95 potential MFS permeases. The latter were classified according to both phylogeny and function based TC system earlier developed by Saier [13,15]. This approach enabled us to cluster these 95 MFS proteins into 17 distinct families. Indeed each of the predictions must be tested experimentally before final conclusions are reached with reference to the expression and function of the proteins analyzed.

**Figure 3:**
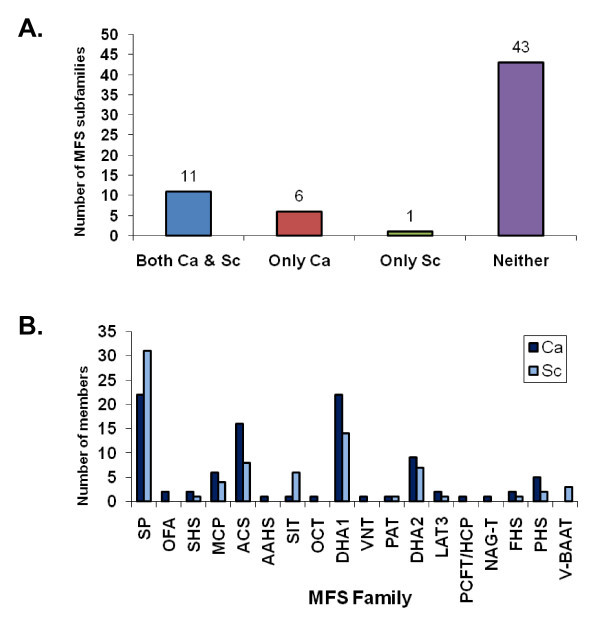
**Distribution of MFS families in *C. albicans *as per transport commission (TC) system and comparison with *S. cerevisiae***. (A) Family designations were according to TC system as mentioned in the *Methods*. Ca and Sc stand for *C. albicans *and *S. cerevisiae*, respectively. Out of 61 reported MFS families in TC database , 17 were identified in *C. albicans *as compared to 12 known in *S. cerevisiae*. (B) A comparison of MFS families between *C. albicans *and *S. cerevisiae *revealed that the members of the same family were almost equal in number in both the yeasts. Interestingly, 6 families that were present in *C. albicans *had no representation in *S. cerevisiae *whereas there was only one such family in *S. cerevisiae *which had no counterpart in *C. albicans*.

The comparison between *C. albicans *and *S. cerevisiae *MFS genes revealed that predominantly most of the families are present in both the organisms (Figure 3A). Notably, there were few families that were present only in *C. albicans*: OFA, AAHS, OCT, VNT, PCFT/HCP and NAG-T (Figure 3B). Interestingly, in OFA family, the *S. cerevisiae *MFS genes, *MHC1 *and YMR155W, although orthologues of *C. albicans *orf19.6180 and orf19.1424, respectively, yet do not conform to the same TC family designation as *Candida*, rather these genes were found to be more similar to OxlT of *Oxalobacter formigenes*, a bacterial oxalate: formate antiporter (Additional file 1). Similar was the case with *C. albicans *orf19.6976, where the *S. cerevisiae *orthologue, YJL163C, belong to DHA1 family instead of PCFT/HCP family (Additional file 1). It was also observed in the AAHS family, *Candida *genes are more closely related to gram-negative bacteria (Additional file 1) as compared to *S. cerevisiae *which probably explains the absence of homologues of this family in *S. cerevisiae*. On a similar pattern, the MFS genes of *C. albicans *belonging to OCT and VNT families are closely related to animals and no significant homologies were detected in *S. cerevisiae *genome. The NAG-T family also did not find any representation in *S. cerevisiae *genome. Taken together, these findings point out that probably these genes had diverged during the course of evolution. Interestingly, vacuolar basic amino acid (V-BAAT) family present in *S. cerevisiae *has no representation in *C. albicans *(Figure 3B). Although the *C. albicans *ORFs, orf19.1308 and orf19.7554 show significant homology to *S. cerevisiae *V-BAAT family members, *VBA1 *and *VBA2*, respectively, they belong to DHA2 family supporting the fact that V-BAAT family is most similar to the DHA2 family.

Our expression analysis of the published work revealed that out of 95 MFS genes, 87 are expressed under either basal (uninduced) or in different specific experimental conditions (Additional file 1). Most of the genes identified in the present investigation are expressed either at mRNA or protein level thus validating our analysis. The expression of the remaining 8 genes was not detected. This would imply that either these genes are expressed under specific growth condition or that they may be candidate pseudogenes. To dissect the role of each putative member of MFS superfamily, an essential part of the process will now involve construction of multiple knockout mutants, which will enable to unravel their role in drug or nutrient transport.

## Methods

Media chemicals were obtained from HiMedia (Mumbai, India). Luria Bertani broth and agar media was purchased from Difco, BD Biosciences, NJ, USA. Taq DNA polymerase, ultra pure deoxyribonucleotides (dATP, dGTP, dCTP and dTTP) were obtained from New England Biolabs (NEB Inc.), USA. Moloney murine leukemia virus (M-MuLV) reverse transcriptases (RT) and RNase inhibitor were obtained from MBI Fermentas. Oligotex mRNA Mini Kit was purchased from Qiagen. Oligonucleotides used were commercially synthesized from Sigma-Aldrich. All Molecular Biology (MB) grade chemicals used in this study were obtained from Sigma Chemical Co. (St. Louis, USA).

### Identification of *C. albicans *MFS transporter genes

*C. albicans *genome assembly version 21  was searched for MFS genes using well known MFS proteins from Swiss-Prot database  as queries in TBLASTN searches [60]. Our initial query dataset had 230 MFS proteins which were used individually to BLAST *Candida *genome. Out of 230 sequences, 38 were chosen which gave significant E-values and were maximally dissimilar among themselves covering diversity of MFS from plants, fungi and mammals. It should also be noted that although a rather relaxed E-value (0.0001) cut-off was used, the observed E-values between the test sequence and the closest query sequence were much below this threshold indicating that the hits obtained were highly significant. The high-scoring segment pairs (HSPs) returned from TBLASTN searches were checked for duplications using an in-house written Perl script and only those that gave the lowest E-value with one or the other sequences from the query set were kept for further analysis. The overlapping HSPs were merged so as to obtain the largest contiguous stretch of nucleotides in the *C. albicans *genome, which had strong sequence homology with the MFS proteins in the query dataset. Since the test sequences always gave significant hits with a number of query sequences the best individual alignments were merged using overlaps. The availability of multiple TBLASTN matches (on account of the large number of query sequences used) made the merging step relatively easy and unambiguous. It also greatly increased the reliability of identifying a true hit and distinguishing it from false positives. The protein sequences were obtained by a six frame translation of the HSPs, using the tool "transeq" from the EMBOSS package  and taking the largest open reading frame (ORF). 95 ORFs identified from *C. albicans *after the initial TBLASTN searches were then pooled with the query dataset of 38 sequences to form a new query dataset and used iteratively for subsequent searches until no new ORFs were obtained. Subsequently, all potential genes were analyzed for MFS domains using the programs ExPASY PROSITE [61], InterPro [62] and Conserved Domain Database at NCBI [63]. Transmembrane domains were predicted using TMHMM .

### Sequence-based functional grouping of *C. albicans *MFS genes

*C. albicans *MFS genes, as identified above, were further subjected to sequence-based classification according to TC system which is based on both functional and phylogenetic information [13,14,64]. Each putative MFS was individually searched against the TCDB. For this purpose the BLAST server at the transporter database  was used with the default settings and E-value cut-off of 1.0 from the given choices of E-values (1000 to 0.0001). It should be noted that here also we chose a rather relaxed E-value cut-off and the potential MFS identified in *Candida *returned much lower E-values with the MFS sequences in the transporter database. To validate the family designations obtained for *C. albicans *using TC system all the known *S. cerevisiae *MFS proteins were also searched against the TC database using the same method as described for *C. albicans*.

### Phylogenetic relationship with *S. cerevisiae*

A systematic search for *S. cerevisiae *homologues of the proteins was done with each *C. albicans *MFS gene by using SGD BLASTP tool .

### Expression analysis of the putative MFS genes

In order to validate the existence of the putative MFS genes in *C. albicans*, expression analysis was done by extensive mining of the data available from the previous genome and proteome-wide studies (Additional file 1) as well as experimentally by RT-PCR.

### Total RNA isolation

Total RNA from *C. albicans *isolate SC5314 was prepared from mid-logarithmically grown phase cells. In a standard preparation, 10 ml of cells, optical density at 600 nm (OD_600_) of 1.0, were pelleted and washed with 10 ml of ice-cold H_2_O and spun at 5000 rpm. The pellet was resuspended in 1.0 ml of TRI^® ^Reagent (Sigma) and 0.3 ml of ice-cold, acid-washed 0.4–0.6 mm diameter glass beads (Sigma, St. Louis, MO, USA) were added and vortexed for 5 min. Chloroform (0.2 ml) without isoamyl alcohol was added and the tubes were shaken vigorously for 15 s. The samples were incubated at room temperature for 15 min, centrifuged at 12,000 × g for 15 min at 4°C. The upper colourless aqueous phase was transferred to a new tube and 0.5 ml of isopropanol was added. The tubes were incubated at room temperature for 10 min, centrifuged at 12,000 × g for 10 min and the pellet washed with 75% ethanol and recentrifuged. The pellet was air dried and resuspended in 100 μl of H_2_O. All the experiments were done with diethyl pyrocarbonate (DEPC) treated H_2_O. DNA free RNA was prepared by treating total RNA with DNase RQ1 (Promega). The OD_260 _and OD_280 _were measured and the integrity of the total RNA was visualized by subjecting 2–5 μl of the sample to electrophoresis through a denaturing 1% agarose/2.2 M formaldehyde gel. The total RNA preparation isolated was stored at -80°C till further use.

### Reverse transcription PCR (RT-PCR)

The nucleotide sequence of the oligonucleotide primers used for the RT-PCR was taken from CGD . Total RNA isolated from SC5314 (as described above) was enriched with poly(A)^+ ^(polyadenylated) mRNA using the Oligotex mRNA Mini Kit protocol (Qiagen) and used subsequently for performing the reverse transcription reaction as described elsewhere [65]. To synthesize cDNA, *ca*. 0.1 μg of poly(A)^+ ^RNA was placed in a 0.5 ml reaction tube with 1 μM of oligo(dT)_18 _anchor primer stock and the volume was adjusted to 11 μl with DEPC treated water. The mixture was incubated for 10 min at 70°C and chilled on ice for 1 min, after which the remainder of the reaction mixture was added from a master mix to the reaction tube in order for each reaction to contain a 1 mM concentration each of dATP, dCTP, dGTP and dTTP; 40 U of RNase inhibitor in a buffer consisting of 50 mM Tris-HCl (pH 8.3), 50 mM KCl, 4 mM MgCl_2 _and 10 mM DTT. After brief mixing, the reaction was incubated for 10 min at 37°C followed by addition of 40U of M-MuLV reverse transcriptase. Finally, the reaction was incubated at 37°C for 60 min and then stopped by heating at 70°C for 10 min followed by chilling it on ice for 1 min. The synthesized cDNA was purified from unincorporated dNTPs, oligo(dT)_18 _anchor primer and proteins by using Oligotex mRNA Mini Kit. Amplification of specific mRNA of each gene was performed using corresponding appropriate dilution of cDNA as template (generally 1:4) and 1 μM of each specific forward and reverse PCR primer as mentioned in Table 1. (*parameters*: initial denaturation of 95°C for 5 min followed by 35 cycles denaturation at 95°C for 15 s, annealing at 55°C for 30 s, elongation at 72°C for 30 s and final extension at 72°C for 10 min). As a positive control, *MDR1 *specific forward MDR1-F and reverse MDR1-R primer (corresponding to positions 1038–1396 in the *MDR1 *genomic sequence) was also used. The negative control (without RT) established that the PCR products generated in the RT-PCR were not due to genomic DNA contamination (data not shown). Resulting RT-PCR products were electrophoresed on a 1.2% agarose gel in 1× TAE.

## Authors' contributions

MG identified and annotated MFS proteins in *C. albicans *and wrote the manuscript. NP and VR facilitated the bioinformatics analyses, particularly with respect to accessing and extracting database information and domain identification. RM was responsible for the experimental part. GM took part in the revision of this article. DC contributed expert knowledge and participated in the design and coordination of the study. RP conceived the study and helped to draft the manuscript. All authors read and approved the final manuscript.

## Supplementary Material

Additional file 1 **A summary of *C. albicans *potential MFS genes listing TC family designation, CGD ORF, gene, alias, TCDB homolog and expression confirmation along with the closest *S. cerevisiae *member within the TC family**. ^a ^Saier's Transport Commission (TC). ^b ^CGD ORF number . ^c ^TCDB homolog obtained from BLAST searches in the transporter database . ^d ^Systematic search for *S. cerevisiae *homologues of the proteins was done with each gene by using SGD BLASTP tool . ^e ^Expression Confirmation – M: Microarray, N: Northern Analysis, R: RT-PCR, MS: Mass Spectroscopy. The data has been analysed from the genome/proteome wide studies on *C. albicans *[16,23,66-71]. Bold, *Italics *ORFs indicate genes which were experimentally tested by RT-PCR in the present study. * Genes with a strong homolog in fungi but absent from human and murine genomes [28]. Click here for file
