# Supplementary material for: MFS transportome of the human pathogenic yeast Candida albicans
Source: BMC Genomics. 2008 Dec 3;9:579. doi: 10.1186/1471-2164-9-579 (PMC2636803; doi:10.1186/1471-2164-9-579)
Supplement: Additional file 1 — A summary of C. albicans potential MFS genes listing TC family designation, CGD ORF, gene, alias, TCDB homolog and expression confirmation along with the closest S. cerevisiae member within the TC family. a Saier's Transport Commission (TC). b CGD ORF number . c TCDB homolog obtained from BLAST searches in the transporter database . d Systematic search for S. cerevisiae homologues of the proteins was done with each gene by using SGD BLASTP tool . e Expression Confirmation – M: Microarray, N: Northern Analysis, R: RT-PCR, MS: Mass Spectroscopy. The data has been analysed from the genome/proteome wide studies on C. albicans [16,23,66-71]. Bold, Italics ORFs indicate genes which were experimentally tested by RT-PCR in the present study. * Genes with a strong homolog in fungi but absent from human and murine genomes [28]. [file 1471-2164-9-579-S1.doc]

**Additional file 1**

| **TC Family^a^** | **CGD ORF^b^** | **Gene** | **Alias** | **TCDB homolog^c^** | ***S. cerevisiae*^d^**  **Homolog** | **Expression Confirmation^e^** |
| --- | --- | --- | --- | --- | --- | --- |
| **2.A.1.1 The Sugar Porter (SP) Family** | | | | | |  |
|  | orf19.5447  orf19.4682  orf19.2633  orf19.6141  orf19.3526  orf19.3981  orf19.5962  orf19.7094  orf19.2023  orf19.2021  orf19.2020  orf19.4384  orf19.5753  orf19.7093  orf19.4923  orf19.4527  orf19.3668  orf19.4356  orf19.6005  orf19.644  orf19.1587  orf19.2425 | *HGT19*  *HGT17*  *HGT14*  *HGT16*  *HGT15*  *MAL31*  *SNF3*  *HGT12*  *HGT7*  *HGT8*  *HGT6*  *HXT5*  *HGT10*  *HGT13*  *HGT1*  *HGT2*  *HGT3*  *HGT5*  *HGT9*  *HGT20*  *HGT18* | *ITR1*  *ITR2*  *GSP1, HGT4*  *SNF32*  *HXT62*  *HGT11, HXT3, HXT7*  *HXT5,HXT4*  *HXT4, HXT61*  *HXT10*  *STL1*  *STL1, STL2*  *RGT2*  *HXT4* | Arabinose:H^+^ symporter  Quinate:H^+^ symporter  Quinate:H^+^ symporter  Quinate:H^+^ symporter  Myoinositol:H^+^ symporter  Maltose:H^+^ symporter  Glucose (high) sensor  Glucose (high) sensor  High affinity glucose uniporter  High affinity glucose uniporter  High affinity glucose uniporter  Fructose:H^+^ symporter  Glycerol:H^+^ symporter  Glycerol:H^+^ symporter  High affinity glucose transporter  High affinity glucose transporter  High affinity glucose transporter  High affinity glucose transporter  Xylose facilitator  Xylose facilitator  Monosaccharide:H^+^ symporter  Monosaccharide:H^+^ symporter | *HXT3*  *HXT17*  *HXT4*  *HXT1*  *ITR2*  *MAL31*  *SNF3*  *SNF3*  *HXT7*  *HXT7*  *HXT7/ HXT6*  *HXT5*  *STL1*  *STL1*  YFL040W  *HXT11*  *HXT11*  YDL199C  *STL1*  *HXT5*  YBR241C  *VPS73* | M,R  R  M,R  M,R  M,R,MS  M,MS  M,R  M,R,MS  M,R  M,R  M,R  M,R  M,R,MS  R  M  M,MS  M,R  M,R  M,R  M,R  M,R  M,R |
| **2.A.1.2 The Drug: H+ Antiporter-1 (12 Spanner) (DHA1) Family** | | | | | |  |
|  | orf19.5604*  orf19.3218  orf19.4737*  orf19.341*  orf19.2158  orf19.3395  orf19.6577*  orf19.7148*  orf19.2160*  orf19.136  orf19.4780  orf19.4550  orf19.6992  orf19.553  orf19.508  orf19.2991  ***orf19.1582***  orf19.2517  orf19.4889  orf19.4546  orf19.3432  orf19.473* | *MDR1*  *TPO3*  *NAG3*  *FLU1*  *TPO2*  *NAG4*  *QDR1*  *HOL1*  *HOL4*  *TPO4* | *BMR1, BEN1*  *DHA12*  *TPO3*  *MDR97, TMP1*  *TPO1*  *TMP2* | Benomyl,Methotrexate,Fluconazole  Quinolone :H^+^ antiporter  Polyamines (spermine, spermidine, putrescene); paraquat; methylgloxal bis(guanylhydrazone):H^+^ antiporter  Fluconazole  Fosfomycin  Fosfomycin  Quinidine, Barban, Cisplatin & Bleomycin  Histidinol & Na^+^ resistance  Fluoroquinolone & other compounds  Caffeine | *FLR1*  YJR124C  *TPO3*  *TPO1*  *TPO3*  YHR048W  *TPO1*  *TPO1*  *TPO3*  *QDR3*  YJR124C  YCR023C  *QDR1*  *DTR1*  *QDR1*  *HOL1*  *HOL1*  *HOL1*  *HOL1*  *HOL1*  YCR023C  *TPO4* | M  M  M,MS  M,N  M  M  M,MS  N  M  M  M  M  M  M  M  M  M  M  M,MS  M  M |
| **2.A.1.3 The Drug: H+ Antiporter-2 (14 Spanner) (DHA2) Family** | | | | | |  |
|  | orf19.304*  orf19.2350  orf19.2923  orf19.1942  orf19.4779*  orf19.3444  orf19.1308  orf19.7554  ***orf19.7336*** | *SGE1* |  | (Aminotriazole, 4-nitroquinoline-N-oxide,etc.):H^+^ antiporter  Me2^+^·tetracycline:2H^+^ antiporter  Vacuolar basic amino acid (Arg, Lys, His) transporter  Metal:tetracycline/oxytetracycline efflux pump | YOR378W  YOR378W  YMR155W  *AZR1*  YKR105C  *AZR1*  *VBA1*  *VBA2*  *AZR1* | M  M  M  M,MS  M,MS  M  M  M |
| **2.A.1.7 The Fucose: H+ Symporter (FHS) Family** | | | | | |  |
|  | ***orf19.4090***  orf19.7490 |  |  | Sucrose  Sucrose | *BSC6*  *BSC6* | M |
| **2.A.1.9 The Phosphate: H+ Symporter (PHS) Family** | | | | | |  |
|  | orf19.655  orf19.1980*  orf19.1979*  orf19.34*  orf19.1978* | *PHO84*  *GIT4*  *GIT1*  *GIT2* | *IFN1 , GIT3*  *GIT1* | Pi (high-affinity) : H^+^ symporter  Pi uptake porter  Pi uptake porter  Pi uptake porter  Pi uptake porter | *PHO84*  *GIT1*  *GIT1*  *GIT1*  *GIT1* | M,MS  M  M,MS  M  M,MS |
| **2.A.1.11 The Oxalate: Formate Antiporter (OFA) Family** | | | | | |  |
|  | ***orf19.6180***  ***orf19.1424*** |  |  | Oxalate:formate antiporter  Oxalate:formate antiporter | *MCH1*  YMR155W |  |
| **2.A.1.12 The Sialate: H+ Symporter (SHS) Family** | | | | | |  |
|  | orf19.7447  orf19.5307 | *JEN1*  *JEN2* |  | Lactate/pyruvate:H^+^ symporter  Lactate/pyruvate:H^+^ symporter | *JEN1*  *JEN1* | M  M |
| **2.A.1.13 The Monocarboxylate Porter (MCP) Family** | | | | | |  |
|  | orf19.6209  orf19.1584  orf19.2751  orf19.4337  orf19.5720  orf19.6263 |  |  | Aromatic aa(Tyr,Trp, Phe) transporter  Riboflavin  Riboflavin  Riboflavin  Riboflavin  Riboflavin | *MCH2*  *MCH5*  *MCH4*  *ESBP6*  *MCH4*  *MCH4* | M  M  M  M  M  M |
| **2.A.1.14 The Anion: Cation Symporter (ACS) Family** | | | | | |  |
|  | orf19.6522*  ***orf19.6520****  orf19.7158*  orf19.5023*  orf19.6956*  orf19.1427  orf19.309*  orf19.5859*  orf19.3208*  orf19.4335*  orf19.3232*  orf19.700  orf19.1855  orf19.7666  orf19.5535  orf19.2397 | *DAL7*  *DAL9*  *DAL5*  *DAL8*  *DAL52*  *TNA1*  *SEO1* | *DAL6*  *SEO2*  *SEO2 , SEO1* | Tartrate  Tartrate  Tartrate  Allantoate  Nicotinate  Nicotinate  Biotin:H^+^ symporter  Biotin:H^+^ symporter  Pantothenate: H^+^ symporter  Pantothenate: H^+^ symporter  Biotin:H^+^ symporter | YIL166C  YIL166C  YIL166C  *DAL5*  *DAL5*  *THI73*  *DAL5*  *DAL5*  *DAL5*  *TNA1*  *TNA1*  *SEO1*  *SEO1*  *SEO1*  *FEN2*  YIL166C | M  M  M  M  M  M  M  M  M,MS  M  M,MS  M  M  M  M,MS |
| **2.A.1.15 The Aromatic Acid: H+ Symporter (AAHS) Family** | | | | | |  |
|  | orf19.6592 |  |  | Niacin |  | M |
| **2.A.1.16 The Siderophore-Iron Transporter (SIT) Family** | | | | | |  |
|  | orf19.2179* | *SIT1* | *ARN1* | Ferrichrome:H^+^ symporter | *ARN1* | M,N,MS |
| **2.A.1.19 The Organic Cation Transporter (OCT) Family** | | | | | |  |
|  | orf19.7071 | *FGR2* | *PHO84* | Organic anion :di-carboxylate |  | M |
| **2.A.1.22 The Vesicular Neurotransmitter Transporter (VNT Family)** | | | | | |  |
|  | orf19.6578 |  |  | Dopamine |  | M |
| **2.A.1.25 The Peptide-Acetyl-Coenzyme A Transporter (PAT) Family** | | | | | |  |
|  | orf19.3782 |  |  | Acetyl-CoA:CoA antiporter | YBR220C | M |
| **2.A.1.44 The L-Amino Acid Transporter-3 (LAT3) Family** | | | | | |  |
|  | ***orf19.6654*** orf19.6316 |  |  | L-amino acids  L-amino acids | YMR221C  YMR221C | M |
| **2.A.1.50 The Proton Coupled Folate Transporter/Heme Carrier Protein (PCFT/HCP) Family** | | | | | |  |
|  | ***orf19.6976*** |  |  | Proton coupled, high affinity folate porter | YJL163C |  |
| **2.A.1.58 The N-Acetylglucosamine Transporter (NAG-T) Family** | | | | | |  |
|  | orf19.5392 | *NGT1* |  | N-acetylglucosamine |  | M,MS |
